# Retraction: Down-regulation of miR-34a-5p potentiates protective effect of adipose-derived mesenchymal stem cells against ischemic myocardial infarction by stimulating the expression of C1q/tumor necrosis factor-related protein-9

**DOI:** 10.3389/fphys.2025.1628944

**Published:** 2025-06-04

**Authors:** Frontiers Editorial Office

The journal retracts the 17 December 2019 article cited above.

Following publication, concerns were raised regarding the integrity of the images in the published figures. The authors failed to provide a satisfactory explanation during the investigation, which was conducted in accordance with Frontiers’ policies. As a result, the data and conclusions of the article have been deemed unreliable and the article has been retracted.

This retraction was approved by the Chief Editors of Frontiers in Physiology and the Chief Executive Editor of Frontiers. The authors have not responded to correspondence regarding this retraction.

